# Various Kinds of Dentition

**Published:** 1892-06

**Authors:** N. E. Root

**Affiliations:** Cleveland, Ohio


### Various Kinds of Dentition.

BY N. E. ROOT, D.D.S., CLEVELAND, OHIO.

Teeth are peculiar to the vertebrate animals and are attached to parts of the mouth, commonly to the jaws. They present many varieties as to number, size, form, structure, position and mode of attachment; but are principally adapted for seizing, tearing, dividing or grinding the food. In some species, teeth serve as weapons of offence and defence, and as aids in locomotion, transportation and procuring of food. They are characteristic of age and sex, and in man, subservient to beauty and speech. Teeth, may be found in fish, reptiles, snakes and mammals generally. The teeth of fish show greater variety than those of any other class of animals, they range in number from zero to countless quantities. In form, they are generally conicle, slender, sharp, pointed and very numerous. In the shark, the teeth are supported by the upper and lower jaws, as in most quadrupeds, but many other fish have teeth growing from the roof of the mouth, from the surface of the tongue, and even from the bones of the nose and base of skull. The mode of attachment is varied, being fibrous and elastic hinge, or by anchylosis or implantation. In all fish, teeth are shed and renewed, not only once as in mammals, but frequently during the whole course of their lives. In the class of reptiles called chelonians, as the tortoise and turtle, they are devoid of teeth.

No species of toads possess teeth, but frogs have teeth in the upper jaw. In the lizard, and most serpents, teeth may be found in both jaws and also upon the palate. In most lizards, teeth are anchylosed to each jaw; and in crocodiles they are implanted in sockets.

In mammals, as in fish and reptiles, there are to be found species that are devoid of teeth. The ant-eaters being an example of this type. Whales have whale-bone substitutes in the upper jaw. The elephant has never more than one entire molar, or parts of two, in use on each side of the upper and lower jaws, to which are added two tusks in the upper jaw. The common number of teeth in rodents is twenty. Whilst hares and rabbits have twenty-eight each. Thirty-two teeth characterizes man, and is the average number of this class of mammals, although the typical number is forty-four. The maximum number of this class is one hundred and ninety, which are found in the true dolphins. the common porpoise has between eighty and ninety, and the armadillo ninety-eight. Where teeth are in excessive number they are small and of a simple conicle form, while in most other mammals, the teeth have special forms for special uses, as is well known in those of man. It is peculiar to the class of mammals to have teeth implanted in sockets, and confined to the maxillary bones, and form a single row in each; they may be present however in only one, but are generally found in both bones.

